# Deciphering von Hippel-Lindau (VHL/Vhl)-Associated Pancreatic Manifestations by Inactivating *Vhl* in Specific Pancreatic Cell Populations

**DOI:** 10.1371/journal.pone.0004897

**Published:** 2009-04-02

**Authors:** H.-C. Jennifer Shen, Asha Adem, Kris Ylaya, Arianne Wilson, Mei He, Dominique Lorang, Stephen M. Hewitt, Klaus Pechhold, David M. Harlan, Irina A. Lubensky, Laura S. Schmidt, W. Marston Linehan, Steven K. Libutti

**Affiliations:** 1 Tumor Angiogenesis Section, Surgery Branch, National Cancer Institute, Bethesda, Maryland, United States of America; 2 Tissue Array Research Program, Laboratory of Pathology, National Cancer Institute, Bethesda, Maryland, United States of America; 3 Diabetes Branch, National Institute of Diabetes and Digestive and Kidney Diseases, Bethesda, Maryland, United States of America; 4 Division of Cancer Treatment and Diagnosis, National Cancer Institute, Rockville, Maryland, United States of America; 5 Urologic Oncology Branch, National Cancer Institute, Bethesda, Maryland, United States of America; 6 Basic Research Program, Science Applications International Corporation (SAIC)-Frederick, Inc., National Cancer Institute-Frederick, Frederick, Maryland, United States of America; Harvard Medical School, United States of America

## Abstract

The von Hippel-Lindau (VHL) syndrome is a pleomorphic familial disease characterized by the development of highly vascularized tumors, such as hemangioblastomas of the central nervous system, pheochromocytomas, renal cell carcinomas, cysts and neuroendocrine tumors of the pancreas. Up to 75% of VHL patients are affected by VHL-associated pancreatic lesions; however, very few reports in the published literature have described the cellular origins and biological roles of VHL in the pancreas. Since homozygous loss of *Vhl* in mice resulted in embryonic lethality, this study aimed to characterize the functional significance of VHL in the pancreas by conditionally inactivating *Vhl* utilizing the Cre/LoxP system. Specifically, *Vhl* was inactivated in different pancreatic cell populations distinguished by their roles during embryonic organ development and their endocrine lineage commitment. With Cre recombinase expression directed by a glucagon promoter in α-cells or an insulin promoter in β-cells, we showed that deletion of *Vhl* is dispensable for normal functions of the endocrine pancreas. In addition, deficiency of VHL protein (pVHL) in terminally differentiated α-cells or β-cells is insufficient to induce pancreatic neuroendocrine tumorigenesis. Most significantly, we presented the first mouse model of VHL-associated pancreatic disease in mice lacking pVHL utilizing Pdx1-Cre transgenic mice to inactivate *Vhl* in pancreatic progenitor cells. The highly vascularized microcystic adenomas and hyperplastic islets that developed in Pdx1-Cre;Vhl f/f homozygous mice exhibited clinical features similar to VHL patients. Establishment of three different, cell-specific *Vhl* knockouts in the pancreas have allowed us to provide evidence suggesting that VHL is functionally important for postnatal ductal and exocrine pancreas, and that VHL-associated pancreatic lesions are likely to originate from progenitor cells, not mature endocrine cells. The novel model systems reported here will provide the basis for further functional and genetic studies to define molecular mechanisms involved in VHL-associated pancreatic diseases.

## Introduction

The von Hippel-Lindau (VHL) syndrome is an autosomal, dominant inherited disorder caused by mutations in the *VHL* tumor suppressor gene. VHL patients are predisposed to develop highly vascular tumors in multiple organs, including hemangioblastomas of the retina and central nervous system (CNS), clear cell renal carcinomas, pheochromocytomas, cyst and neuroendocrine tumors in the pancreas [1]. This familial cancer syndrome is caused by germ-line mutations in the *VHL* gene, which was mapped to chromosome 3p25 by positional cloning [2]. Following Knudson's two-hit hypothesis, loss or inactivation of the remaining wildtype allele is associated with VHL tumorigenesis [3], [4]. The spectrum of VHL tumors in affected families varies [5] and biochemical analysis of the *VHL* gene product has provided the molecular basis that explains the phenotype-genotype correlations evident in VHL disease [6], [7], [8], [9].

At the molecular level, the von Hippel-Lindau protein (pVHL) is a critical factor in the oxygen sensing pathway. Under normoxic conditions, pVHL forms a multiprotein complex with E3 ubiquitin ligase that targets the α-subunits of hypoxia-inducible factor (HIFα) for degradation by the proteosome [10], [11]. Under hypoxic conditions, HIFα subunits escape ubiquitin-mediated proteolysis, allowing HIFα to accumulate, translocate to the nucleus, and activate downstream targets. In subsets of VHL mutations, the lack of functional pVHL leads to accumulation of HIF, and results in the activation of HIFα target genes even in the presence of oxygen [12]. Enhanced transcription of a wide variety of HIFα target genes, such as vascular endothelial growth factor (VEGF), is thought to contribute to the highly vascular tumors that develop in VHL patients [13]. Independent of its function in regulating the HIFα pathway, pVHL also binds other cellular proteins [14], [15], [16] and promotes extracellular matrix assembly [16], [17]. For example, pVHL has been shown to bind and stabilize microtubule structures [18], and regulate fibronectin deposition to maintain vascular integrity [19], [20].

Expression of *VHL* has been reported in most tissues and cell types, but little is known about its role in normal development [2], [21], [22]. Homozygous inactivation of *Vhl* in mice resulted in embryonic lethality at embryonic stage (E) E10.5–E12.5 due to defects in placental vasculogenesis [23]. Thus, tissue-specific knockout of *Vhl* has been utilized to investigate the biological functions of pVHL. Inactivation of *Vhl* utilizing an albumin-cre in liver or a mosaic β-actin-cre resulted in hepatic vascular tumors [24], [25], similar to the ones observed in *Vhl* heterozygous knockout animals. Unexpected roles of *Vhl* in spermatogenesis, thymus cell survival, and bone development have also been reported with conditional loss of pVHL [25], [26], [27]. Notably, inactivation of *Vhl* in renal epithelial cells led to development of tubular cysts that share morphologic and molecular characteristics with renal cysts found in VHL patients [28]. While genetic analysis of *Vhl* in mice have uncovered novel functions of pVHL in various tissues, much is yet to be learned about pVHL's role in organs normally affected in VHL patients, such as CNS, adrenals and pancreas.

In VHL patients, the most common manifestations affecting the pancreas are benign cysts and microcystic adenomas (MCA), which occur in 35–75% of VHL patients [29], [30]. In addition, 12–17% of VHL patients develop pancreatic neuroendocrine (islet cell) tumors, which possess the malignant potential to develop metastasis [31], [32]. However, the cellular origins and molecular mechanisms leading to these pancreatic lesions found in VHL patients are not known. Here we present three mouse models of VHL in the pancreas generated by conditionally inactivating *Vhl* in specific pancreatic cell populations. Not previously reported in the literature, we showed that pVHL is dispensable for the normal functions of terminally differentiated islet α-cells and β-cells in the endocrine pancreas. Whereas mice with *Vhl* deletion in endocrine pancreas showed normal survival, mice lacking pVHL in pancreatic progenitor cells that give arise to both exocrine and endocrine pancreas (Pdx1-Cre;Vhl f/f), exhibited significant postnatal death. Most significantly, the few surviving Pdx1-Cre;Vhl f/f mice developed MCA and islet hyperplasia, similar to those found in VHL patients. Taken together, these results provided evidence suggesting that pVHL is functionally important for postnatal exocrine pancreas, and that VHL-associated pancreatic lesions originate from progenitor cell lineages. The novel model systems reported here will provide the basis for further genetic studies to define molecular events involved in VHL-associated pancreatic diseases.

## Results

### Loss of pVHL in α-cells is Dispensable and Insufficient to Induce Tumorigenesis

**Figure 1:**
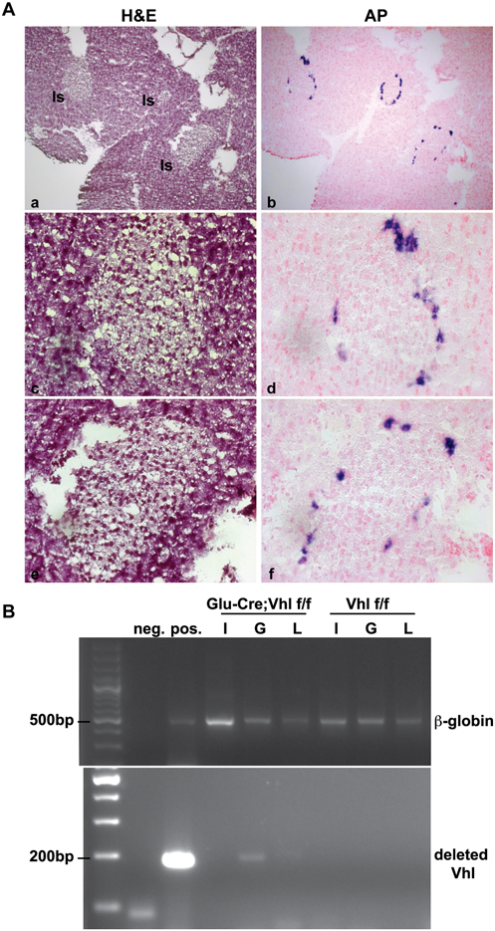
Analysis of Glu-Cre;Vhl colony. A. Validation of the Glu-Cre transgenic line with Z/AP reporter mice. H&E (panels a, c, e) and alkaline phosphatase (AP; panels b, d, f) staining of a representative Glu-Cre;Z/AP pancreas at 5 months of age. Expression of Glu-Cre localized in α-cells at the outer ring of endocrine islets, as indicated by purple AP stain. Pancreatic islets (Is) are as indicated in panel a, and images are shown at 100× (a, b) and 400× (c, d, e, f) magnification. B. Genotyping PCR using genomic DNA isolated from different cell populations of the endocrine pancreas in Glu-Cre;Vhl f/f and Vhl f/f mice at 27 months of age. Insulin-positive β-cells (I), glucagon-positive α-cells (G), and lectin-positive endothelial cells (L) were collected via flow cytometry. Negative (neg.) and positive (pos.) controls are shown next to the ladder (far left lane). Top panel shows the PCR for *β-globin* alleles to demonstrate the presence of genomic DNA for each cell population. Bottom panel indicates the presence of deleted *Vhl* alleles only in α-cells of Glu-Cre;Vhl f/f pancreas.

VHL-associated pancreatic neuroendocrine tumors (PNET) afflict 12–17% of VHL patients, and these tumors have been demonstrated to exhibit focal positivity for glucagon [33]. Thus, we speculated that these PNET might originate from glucagon-positive α-cells deficient of pVHL in the endocrine pancreas. To inactivate *Vhl* specifically in α-cells, we first generated an α-cell specific Cre transgenic line (Glu-Cre) utilizing a rat glucagon promoter sequence to direct Cre recombinase expression. By crossing a Glu-Cre transgenic line with the Z/AP reporter mice [34], we confirmed α-cell specific expression of Cre recombinase in Glu-Cre;Z/AP pancreas at 5 and 12 months of age. As expected, expression of Cre-recombinase localized in α-cells at the outer ring of endocrine islets, as indicated by purple alkaline phosphatase (AP) staining (Figure 1A, right panels). No positive AP expression in pancreas was observed in age-matched control genotypes of Glu-Cre and Z/AP mice (data not shown).

To determine the functional significance of *Vhl* in α-cells, the Glu-Cre transgenic mice were next bred with mice in which exons 2 and 3 of the *Vhl* alleles are flanked by loxP sites (flox or f) [25]. Since variability in phenotype was documented in other tissue-specific knockouts of *Vhl* [24], [25], we generated *Vhl*-deficient α-cells in A/J and C57BL/6 backgrounds. At the time of weaning (3–4 weeks after birth), we obtained the expected Mendelian frequencies for all genotypes in both background strains (Table 1). Mouse pancreases with *Vhl* deletion in α-cells (Glu-Cre;Vhl f/f, n = 12) appeared histological normal when compared to age-matched control genotypes (Glu-Cre, n = 8 and Vhl f/f, n = 6) from 10 months to 23 months of age (data not shown). No obvious physiological and behavioral phenotypes were observed for Glu-Cre;Vhl f/f mice. To confirm that *Vhl* alleles were indeed deleted in Glu-Cre;Vhl f/f mice, we isolated α-cells, β-cells and endothelial cells from pancreatic islets via flow cytometry, and obtained genomic DNA for each cell population. Genotyping PCR analysis showed the expected deletion of *Vhl* alleles only in glucagon-positive α-cells, but not in insulin-positive β-cells and lectin-positive endothelial cells in Glu-Cre;Vhl f/f animals at 27 months of age (Figure 1B). Together, these results demonstrated that *Vhl* is not essential for mature α-cells, and that deficiency of *Vhl* in α-cells is insufficient to cause tumorigenesis in the endocrine pancreas.

**Table 1:** Expected Mendelian distribution in Glu-Cre;Vhl colony.

Glu-Cre positive offspring						
strain	Cre;Vhl +/+	Cre;Vhl f/+	Cre;Vhl f/f	N	χ^2^	p-value
**A/J**	30.6%	46.9%	22.4%	49	0.837	0.658
**C57BL/6**	29.1%	40.5%	30.4%	79	2.873	0.238
Mendelian %	25.0%	50.0%	25.0%			

Glu-Cre negative offspring						
strain	Vhl +/+	Vhl f/+	Vhl f/f	N	χ^2^	p-value
**A/J**	23.1%	30.8%	46.2%	13	3.308	0.191
**C57BL/6**	28.6%	53.6%	17.9%	28	0.786	0.675
Mendelian %	25.0%	50.0%	25.0%			

Glu-Cre;Vhl f/+ mice were intercrossed and the genotypes of their offspring determined for each background strain at time of weaning.

### Vhl is not essential for Normal Function of Differentiated Islet β-cell

Since β-cells are the major cell type in the endocrine pancreas and some VHL PNETs are positive for insulin staining [33], we next hypothesized that *Vhl* deficiency in insulin-positive β-cells might lead to PNET development. To conditionally inactivate *Vhl* in β-cells, we crossed Rip-Cre transgenic mice [35] with mice in which exons 2 and 3 of the *Vhl* alleles are flanked by loxP sites (flox or f). Similar to our findings in the Glu-Cre;Vhl colony, mice from the Rip-Cre;Vhl colony were generated at the expected Mendelian frequencies for all genotypes regardless of the strain backgrounds (Table 2). Histological analysis of Rip-Cre;Vhl f/f mouse pancreases (n = 4) did not show any abnormality or islet tumor formation when compared to age-matched control genotypes (Rip-Cre and Vhl f/f) at 15 months of age (Figure S1A). Immuno-staining of endocrine markers, such as insulin and glucagon, did not reveal distinct features unique to mice deficient for pVHL in β-cells (data not shown). Determination of *Vhl* allele status in the Rip-Cre;Vhl f/f pancreas utilizing DNA isolated from microdissection further confirmed that *Vhl* deletion only occurred in the expected endocrine islets, but not in the surrounding exocrine pancreas (Figure S1B).

**Table 2:** Expected Mendelian distribution in Rip-Cre;Vhl colony.

Rip-Cre positive offspring						
strain	Cre;Vhl +/+	Cre;Vhl f/+	Cre;Vhl f/f	N	χ^2^	p-value
**A/J**	29.3%	56.0%	14.7%	75	4.307	0.116
**C57BL/6**	28.9%	53.9%	17.1%	76	2.605	0.272
**Balb/C**	30.4%	53.6%	16.1%	56	2.571	0.276
Mendelian %	25.0%	50.0%	25.0%			

Rip-Cre negative offspring						
strain	Vhl +/+	Vhl f/+	Vhl f/f	N	χ^2^	p-value
**A/J**	34.2%	50.0%	15.8%	38	2.579	0.275
**C57BL/6**	29.4%	41.2%	29.4%	17	0.529	0.767
**Balb/C**	30.3%	57.6%	12.1%	33	2.939	0.230
Mendelian %	25.0%	50.0%	25.0%			

Rip-Cre;Vhl f/+ mice were intercrossed and the genotypes of their offspring determined for each background strain at time of weaning.

**Figure 2:**
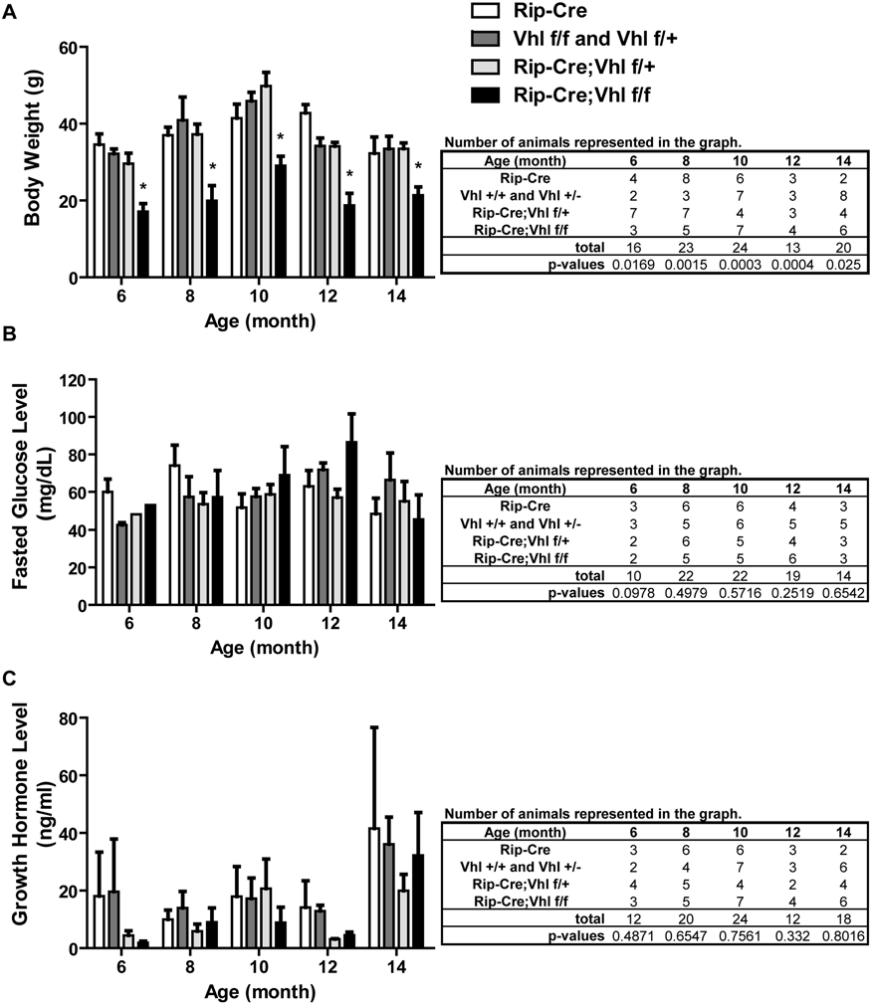
Physiological analyses of control and Rip-Cre;Vhl f/f mice. A. Body weight, B. Fasted glucose levels, and C. Serum growth hormone levels of mice in the Rip-Cre;Vhl colony. Genotypes are as indicated and numbers of animals analyzed at each time point are shown in tables. Data from three different strain backgrounds are combined and shown graphically.

Notably, the physical appearance of the Rip-Cre;Vhl f/f mice were proportionally smaller than their littermates at time of weaning. Indeed, data confirmed that Rip-Cre;Vhl f/f mice weighed significantly less than age-matched littermates at all time points analyzed (Figure 2A). Since *Vhl* has been shown to regulate glucose metabolism [36], we next measured fasting glucose levels in a cohort of Rip-Cre;Vhl mice to determine if an elevated basal glucose level was responsible for the small size seen in these mice. However, our data demonstrated no significant differences in basal glucose levels between control genotypes and Rip-Cre;Vhl f/f mice at all time points measured (Figure 2B). We then speculated that low expression of Cre-recombinase in the hypothalamus reported for Rip-Cre transgenic mice [35] affects hormone feedback loops regulated by the hypothalamus due to inactivation of *Vhl*. Among various hormones regulated by the hypothalamus, we chose to measure growth hormone since its deficiency is associated with lean body mass. Again, our data did not show significant differences in serum growth hormone levels between control genotypes and Rip-Cre;Vhl f/f mice at all time points tested (Figure 2C). Therefore, it is unclear if the small size of Rip-Cre;Vhl f/f mice is due to the loss of *Vhl* in pancreatic islet β-cells or in the hypothalamus.

### Vhl is Functionally Important for Postnatal Exocrine Pancreas

The most common manifestations of pancreatic VHL disease are benign cysts and microcystic adenoma, which occur in 35–75% of VHL patients [29], [30]; however, it remains unclear whether these lesions arise from the endocrine or exocrine pancreas. To investigate whether loss of *Vhl* in the exocrine pancreas can lead to cyst development, we utilized pancreatic and duodenal homeobox 1 (Pdx1) transgenic mice in which cre recombinase expression was driven by the Pdx1 promoter [37]. By breeding Pdx1-Cre transgenic mice with *Vhl* floxed mice, we generated mice with *Vhl* deletion in pancreatic endocrine and exocrine cells as a result of Pdx1-Cre expression in pancreatic progenitor cells during embryogenesis [37]. In contrast to mice deficient in *Vhl* in α-cells or β-cells, we observed some lethality in Pdx1-Cre;Vhl f/f mice. At the time of weaning, the distribution of genotypes was significantly different from the expected values with fewer than expected Pdx1-Cre;Vhl f/f mice in all three different genetic backgrounds (Table 3). Statistically, observed lethality is more severe in the A/J and Balb/C genetic backgrounds than in the C57BL/6 background.

**Table 3:** Lethality in generating Pdx1-Cre;Vhl f/f in A/J and Balb/C backgrounds.

Pdx1-Cre positive offspring						
strain	Cre;Vhl +/+	Cre;Vhl f/+	Cre;Vhl f/f	N	χ^2^	p-value
**A/J**	18.6%	76.7%	4.7%	43	13.977	**0.001**
**C57BL/6**	37.3%	47.5%	15.3%	59	5.881	**0.053**
**Balb/C**	31.5%	64.8%	3.7%	54	13.074	**0.001**
Mendelian %	25.0%	50.0%	25.0%			

Pdx1-Cre negative offspring						
strain	Vhl +/+	Vhl f/+	Vhl f/f	N	χ^2^	p-value
**A/J**	33.3%	45.8%	20.8%	24	0.917	0.632
**C57BL/6**	22.2%	55.6%	22.2%	18	0.222	0.895
**Balb/C**	22.7%	50.0%	27.3%	22	0.091	0.956
Mendelian %	25.0%	50.0%	25.0%			

Pdx1-Cre;Vhl f/+ mice were intercrossed and the genotypes of their offspring determined for each background strain at time of weaning.

**Figure 3:**
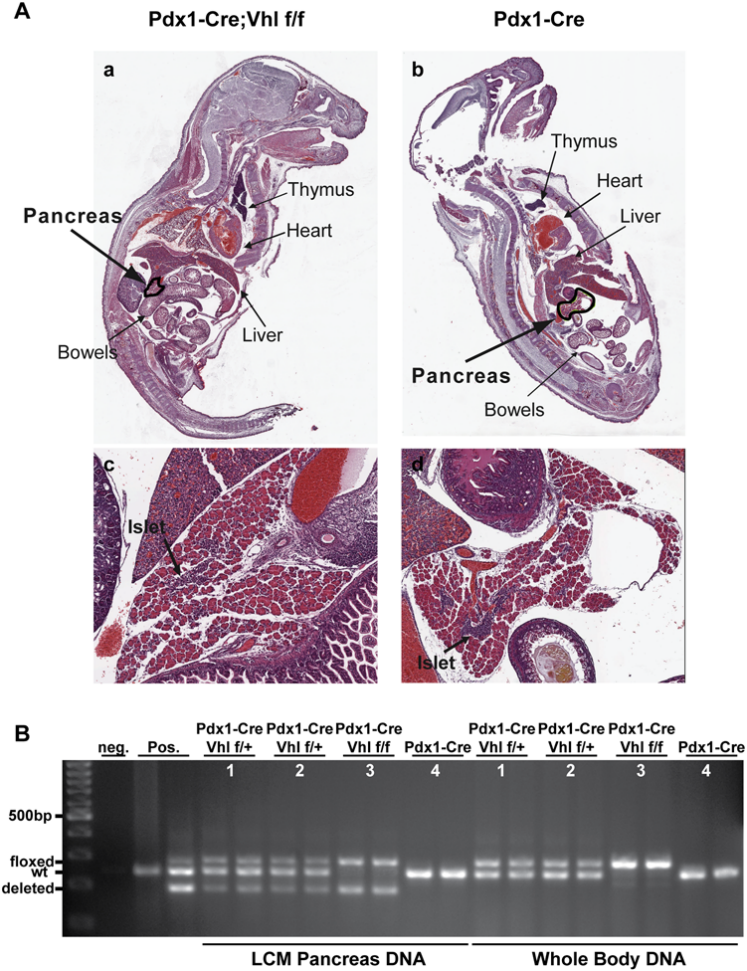
Histological and molecular analyses of control and Pdx1-Cre;Vhl f/f pup pancreas. A. H&E staining of representative Pdx1-Cre, Vhl f/f (panels a and c) and control Pdx1-Cre (panels b and d) mouse pups at postnatal day 3 (P3). Magnified (50×) pup pancreas are shown (panels c and d), and islets are indicated by arrows. B. PCR analysis of *Vhl* allele status. DNA isolated from whole pup section (whole body DNA) and from pancreas via laser capture microdissection (LCM pancreas DNA) was used to detect *Vhl* allele status (floxed, wildtype-wt, deleted). Genotyping PCR was performed in duplicate for each pup (#1–#4). PCR results for Pdx1-Cre;Vhl f/f (#3)and Pdx1-Cre (#4) are the same mouse pups shown in A.

To determine if the lethality in Pdx1-Cre;Vhl f/f mice occurs embryonically or postnatally, Mendelian ratios of the six possible genotypes were observed immediately after birth and during different postnatal time intervals (Table 4). The Pdx1-Cre;Vhl f/f mice were born at the anticipated Mendelian ratio; however, these mice could not survive more than 5 days postnatally (P5). We further analyzed whole-mount histology of mouse pups (P1–P5) in an attempt to uncover the cause of death. However, pathologists blinded for genotypes could not distinguish Pdx1-Cre;Vhl f/f pups from pups of control genotypes (Figure 3A). To confirm that the *Vhl* alleles were indeed deleted in the pancreas of Pdx1-Cre;Vhl f/f newborn pups, whole pup body DNA and pancreatic DNA were isolated by laser capture microdissection. Genotyping analysis demonstrated the expected *Vhl* deleted allele in the Pdx1-Cre;Vhl f/f pup pancreas, as well as the floxed *Vhl* allele in cells of non-pancreatic lineage, such as epithelial and endothelial cells (Figure 3B, animal #3). Thus, whereas mice with homozygous inactivation of *Vhl* in α-cells or β- cells (Glu-Cre;Vhl f/f and Rip-Cre;Vhl f/f, respectively) showed normal survival rates, Pdx1-Cre;Vhl f/f mice exhibited reduced postnatal survival that is likely to be associated with exocrine dysfunction due to the loss of *Vhl* in the exocrine pancreas.

**Table 4:** Postnatal death in Pdx1-Cre;Vhl f/f mice.

Pdx1-Cre positive offspring						
Postnatal (P) days	Cre;Vhl +/+	Cre;Vhl f/+	Cre;Vhl f/f	N	χ^2^	p-value
**P1 (birth)**	5.3%	63.2%	31.6%	19	3.947	0.139
**P3–P5**	22.6%	38.7%	38.7%	31	3.194	0.203
**P7–P10**	31.3%	68.8%	0.0%	16	5.375	0.068
**P21 (weaning)**	25.8%	70.1%	4.1%	97	24.77	**4.2E-06**

Pdx1-Cre negative offspring						
Postnatal (P) days	Vhl +/+	Vhl f/+	Vhl f/f	N	χ^2^	p-value
**P1 (birth)**	12.5%	50.0%	37.5%	8	1.000	0.607
**P3–P5**	14.3%	57.1%	28.6%	7	0.429	0.807
**P7–P10**	60.0%	40.0%	0.0%	10	7.600	0.022
**P21 (weaning)**	28.3%	47.8%	23.9%	46	0.261	0.878

Table 4 combines data from A/J and Balb/C background strains and genotypes were determined at different postnatal (P) time points.

### Loss of Vhl in Exocrine Pancreas Recapitulates Cysts and Microcystic Adenoma (MCA) That Develop in VHL Patients

**Figure 4:**
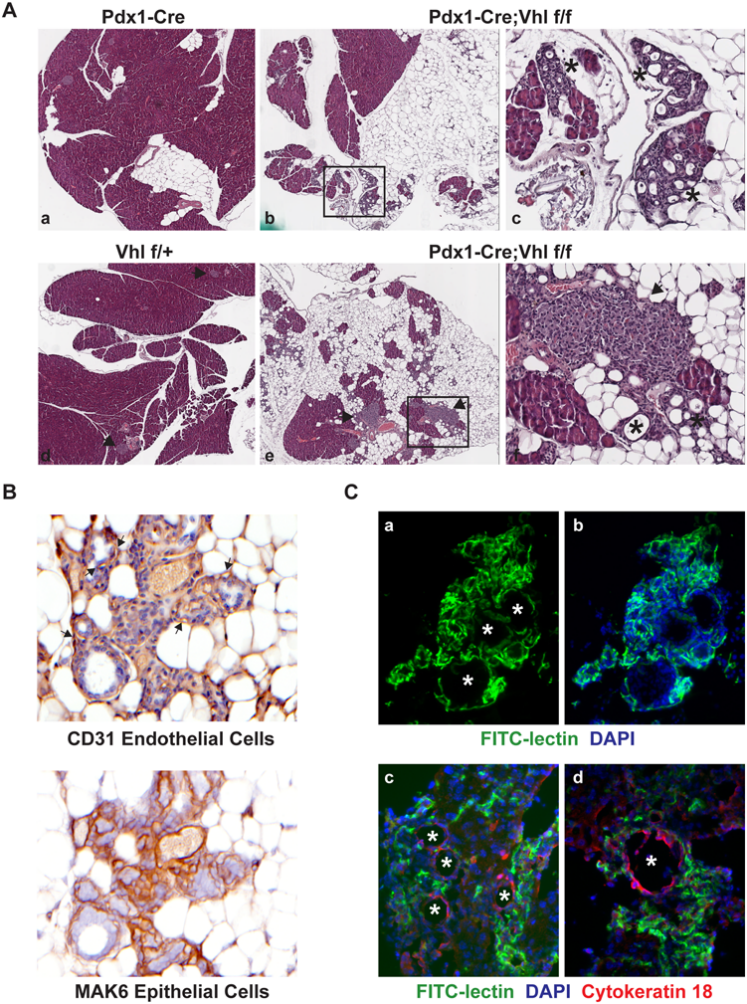
Histological analysis of the microcystic adenomas (MCA) which developed in Pdx1-Cre;Vhl f/f adult pancreas. A. H&E staining of pancreas from representative control Pdx1-Cre (panel a), and Vhl f/+ (panel d), and two different Pdx1-Cre;Vhl f/f (panels b, c, e and f) mice at 16–18 months of age. Gross pancreas histology is shown at 40× in panels a, b, d, e., and areas (black boxes) in panels b and e are magnified at 200× in panels c and f. Islets are indicated by arrows and MCA are indicated by asterisk. B. Immuno-histochemical staining of MCA utilizing a CD31 antibody (top panel) and a pan-keratin MAK6 antibody (bottom panel) to identify endothelial cells and epithelial cells of MCA, respectively. Arrows indicate CD31 positive endothelial cells. Images shown are at 500× magnification. C. Representative immuno-fluorescent images of MCA, indicated by asterisks. FITC-lectin identifies endothelial cells, DAPI identifies cell nuclei and cytokeratin 18 identifies epithelial cells within MCA. Top panels are at 200× and bottom panels are at 400× magnification.

Since the postnatal lethality observed for Pdx1-Cre;Vhl f/f did not exhibit 100% penetrance, we were able to perform histological analysis of the pancreases of the few surviving Pdx1-Cre;Vhl f/f at adulthood in the C57BL/6 background. At 6–7 months of age, Pdx1-Cre;Vhl f/f mouse pancreases (n = 2) were indistinguishable from control genotypes (data not shown). However, at 16–18 months of age, an obvious loss of exocrine pancreas and fat replacement were observed in Pdx1-Cre;Vhl f/f mice (n = 3, Figure 4A). Pathologists blinded for mouse genotypes further identified the presence of cysts and MCA in all Pdx1-Cre;Vhl f/f mice lacking pVHL in the pancreas (Figure 4A, panel c and f).

In VHL patients, the pancreatic cysts and MCA are highly vascularized and characterized by a layer of epithelial lining intermixed with endothelial cells forming capillaries around the cysts [38]. To investigate the similarity between the MCA developed in Pdx1-Cre;Vhl f/f pancreas and that found in VHL patients, we used a CD31 antibody to identify endothelial cells, and a cocktail of cytokeratin antibodies (MAK6) to identify the epithelial cell lining of MCA. Immuno-histochemical staining revealed that epithelial lining of the MCA was positive for MAK6 but not CD31, and that CD31 positive endothelial cells were intermixed within MCA (Figure 4B). The extensive vasculature network of MCA was more evident in Pdx1-Cre;Vhl f/f mice injected with FITC-lectin prior to euthanizing (Figure 4C, panels a and b). Utilizing immuno-fluorescent staining, we further demonstrated that cytokeratin-positive epithelial lining and lectin-positive vasculature did not co-localize, but instead were distinctive cellular structures displayed by MCA in Pdx1-Cre;Vhl f/f animals (Figure 4C, panels c and d).

**Figure 5:**
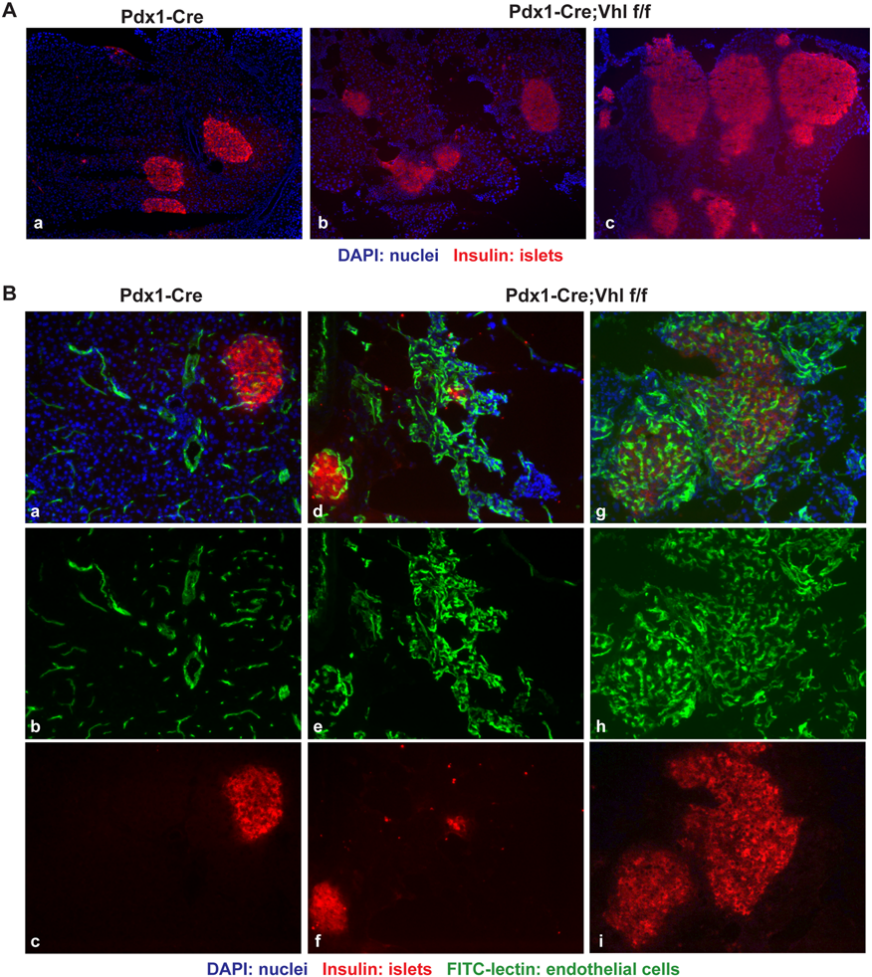
Histological analysis of the endocrine pancreas in Pdx1-Cre;Vhl f/f mice. A. Immuno-fluorescent staining of representative pancreas in Pdx1-Cre (panel a) and Pdx1-Cre;Vhl f/f mice (panels b and c) to demonstrate the abnormally shaped and hyperplastic islets (red) in Pdx1-Cre;Vhl f/f mice at 16–18 months of age. Images are taken at 100×. B. Immuno-fluorescent images of representative pancreas in Pdx1-Cre (panels a–c) and Pdx1-Cre;Vhl f/f (panels d–i) to demonstrate hypervascularity within islets of Pdx1-Cre;Vhl f/f mice. Blood vessels are visualized via FITC-lectin injection (green; panels b, e, and h) while pancreatic islets are identified using an anti-insulin antibody (red; panels c, f, and i). Images are taken at 200×.

Within the pancreas tissue largely replaced by fat deposition, some islets in Pdx1-Cre;Vhl f/f animals appeared small and abnormally shaped without much exocrine acinar cells surrounding them (Figure 4A, panel f), whereas some appeared hyperplastic (Figure 5A). Regardless of the size, islets of Pdx1-Cre;Vhl f/f were characterized by disorganized, dilated and tortuous vascular networks within, as well as outside the islets (Figure 5B). Since VHL neuroendocrine tumors are highly vascularized, it is possible that these islets lacking pVHL might be progressing toward developing pancreatic neuroendocrine tumors.

### Modest Upregulation of Hif1α is Associated with Pancreatic Phenotypes Developed in Pdx1-Cre;Vhl f/f Mice

**Figure 6:**
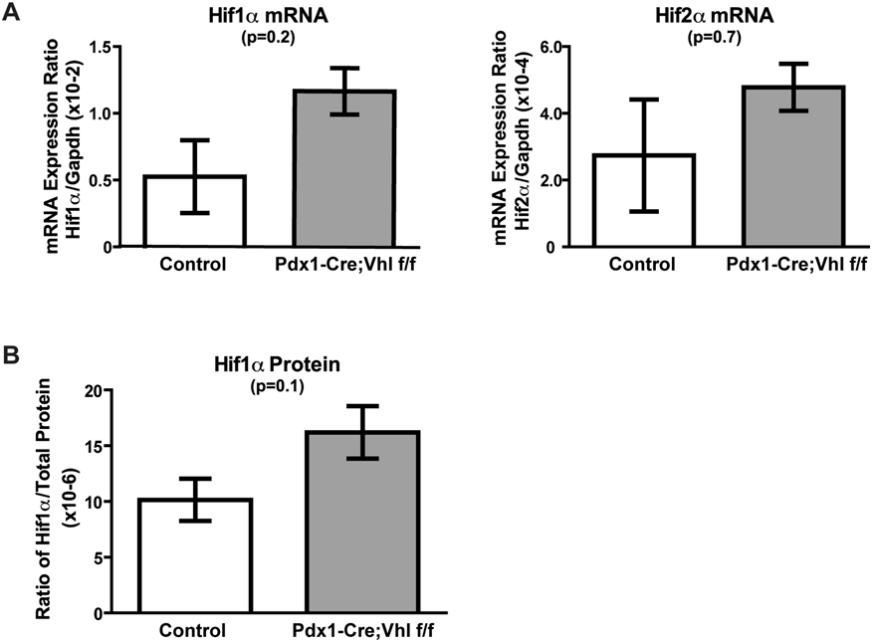
Expression analysis in Pdx1-Cre;Vhl f/f pancreas. Relative gene expression of Hif1α and Hif2α mRNA transcripts (A) and protein expression of Hif1α (B) in pancreas of control (Pdx1-Cre and Vhl f/+), and Pdx1-Cre;Vhl f/f genotypes at older than 16 months of age. Each column represents the average expression of three mice for the indicated genotype.

pVHL deficiency is known to result in constitutive HIF α-subunits stabilization and increased expression of HIF target genes [11]. To examine if the pancreatic phenotypes observed in Pdx1-Cre;Vhl f/f mice were associated with activation of Hif1α or Hif2α pathways, quantitative real-time PCR analysis was performed to detect *Hif1α* and *Hif2α* mRNA expression. As shown in Figure 6A, expression of *Hif1α* was upregulated in Pdx1-Cre;Vhl f/f pancreas (n = 3) when compared with control mice (n = 3). Expression of *Hif2α* and a *Hif2α*-preferred target gene (*Vegf*) [39] appeared similar between control and Pdx1-Cre;Vhl f/f mice (Figure 6A, data not shown). While the upregulation of *Hif1α* mRNA in Pdx1-Cre;Vhl f/f pancreas was not statistically significant, we speculated that this might be the reason why a few of the Pdx1-Cre;Vhl f/f mice survived postnatal lethality. Consistent with the mRNA data, we further confirmed the trend that protein expression of Hif1α was upregulated in the pancreas of pVHL deficient mice utilizing a Hif1α ELISA assay (Figure 6B).

## Discussion

The major significance of this study is that generation of three new pancreatic mouse models of VHL has allowed us to address the cellular origins and biological roles of VHL during pancreatic development and tumorigenesis. In terms of VHL's role during pancreatic development, we demonstrated that deletion of *Vhl* in endocrine α-cells and β-cells which terminally differentiated around E12.5 [40], does not appear to affect normal functions of endocrine pancreas during embryogenesis, postnatal development, and in adulthood (Glu-Cre;Vhl f/f and Rip-Cre;Vhl f/f animals). In contrast, deletion of *Vhl* in pancreatic progenitor cells that give arise to ductal, exocrine and endocrine pancreas resulted in significant postnatal death in mice (Pdx1-Cre;Vhl f/f animals), even though pancreatic organogenesis was not affected during embryonic development. Together, this data suggest that *VHL* is functionally important in postnatal ductal and exocrine pancreas, and that pVHL is not essential for mature endocrine pancreatic cells. While this finding uncovers the novel aspect of *VHL* in postnatal ductal and exocrine pancreas, it remains to be determined whether HIF-dependent or HIF-independent pathways are involved.

In terms of VHL's role during pancreatic tumorigenesis, little is known about the cellular origins and molecular mechanisms related to VHL-associated pancreatic diseases, even though a large portion of VHL patients are affected by pancreatic manifestations, such as cysts, MCA and neuroendocrine tumors. By specifically inactivating *Vhl* in distinct pancreatic cell populations, we reported the first mouse model of VHL that recapitulates some of the clinical features found in the pancreas of VHL patients, and demonstrated that inactivation of *Vhl* in the endocrine pancreas is insufficient to initiate tumorigenesis. VHL-associated pancreatic lesions were only found in mice where *Vhl* was inactivated in pancreatic progenitor cells (Pdx1-Cre;Vhl f/f mice), but not in mice in which *Vhl* was inactivated in mature endocrine cells (Glu-Cre;Vhl f/f and Rip-Cre;Vhl f/f mice). Thus, our data supports the notion of a progenitor cell origin for these VHL-associated pancreatic lesions. In addition, it has been previously suggested by histopathological studies that MCA originated from pancreatic exocrine cells, such as the centroacinar cells [41], [42] or ductal cells [43], [44]. Moreover, none of the VHL patients (n>108) with PNET disease evaluated at our center had a functional neoplasm by hormone levels or symptoms [45]. Together, these data suggest that VHL-associated MCA and PNET originate from pancreatic ductal or exocrine progenitor cells, not differentiated endocrine cells. Analogous to our hypothesis, VHL-associated hemangioblastomas have been demonstrated to derive from embryologic multipotent cells [46]. However, further studies will be required to definitively determine if these VHL-associated pancreatic manifestations can be recapitulated when *Vhl* is specifically deleted in progenitor cells of ductal, exocrine or endocrine lineage.

Similar to pervious reports on *Vhl* knockout mice [24], [25], we also observed variability in phenotypic penetrance of the *Vhl* deletion due to differences in mouse genetic backgrounds. The postnatal lethality in Pdx1-Cre;Vhl f/f mice was most severe in A/J and Balb/C background strains, but not as severe in the C57BL/6 background strain. This finding suggests that strain-specific genetic modifiers may provide protection for the survival of these mice with pVHL deficiency in the pancreas. Notably, we showed that pancreatic phenotypes observed in Pdx1-Cre;Vhl f/f mice correlate with a modest upregulation of Hif1α, but not HIf2α. It is thus possible that these strain-specific genetic modifiers may function to interfere with *VHL* downstream signaling, and compensate for the loss of pVHL. This does not exclude the possibility that the pancreatic phenotypes observed in Pdx1-Cre;Vhl f/f mice resulted from mechanisms completely independent of HIF signaling pathways, such as VHL's role in regulating extracellular matrix assembly [47]. Identification of these modifier genes and further comparative functional analysis utilizing mice deficient in Hif1α and Hif2α will provide insight into the precise molecular mechanisms leading to the development of VHL-associated pancreatic disease.

An unexpected observation during our studies was the small size seen in mice deficient in pVHL in the islet β-cells. While our analyses excluded the idea that alterations in growth hormone and basal glucose levels led to this phenotype in Rip-Cre;Vhl f/f mice, it is described in the literature that pVHL regulates glucose metabolism in liver and kidney cells [10], [36]. Establishment of our Rip-Cre;Vhl f/f homozygous mice now allows us to begin investigating whether or not pVHL also plays an important role in glucose sensing in pancreatic islet β-cells, such as via glucose transporter 1 (Glut1). However, phenotypes seen in Rip-Cre;Vhl f/f mice are confounded by the fact that Rip-Cre is expressed at a low level in the hypothalamus [35]. Thus, it would be critical to confirm and determine that deletion of *Vhl* indeed occurred in particular nuclei within the hypothalamus in Rip-Cre;Vhl f/f homozygous mice. Only when that information is available, will we be able to conclusively interpret the physiological data resulting from the loss of pVHL in pancreatic islet β-cells or in the hypothalamus.

In summary, to decipher the functional significance of pVHL in the pancreas during development and tumorigenesis, we conditionally inactivated *Vhl* in distinct pancreatic cell populations and reported the first mouse model of VHL that recapitulates clinical features found in the pancreas of VHL patients. Importantly, our data demonstrated that pVHL is functionally important for postnatal ductal and exocrine pancreas, and suggested that pancreatic progenitor cells, not mature endocrine cells, as the cell of origin for VHL-associated pancreatic lesions. The novel mouse model systems reported in this study will provide the foundation for further functional and genetic analysis to advance our understanding of VHL-associated pancreatic manifestations.

## Materials and Methods

### Ethics Statement

National Cancer Institute (NCI) and NCI-Frederick are accredited by AAALAC International and follows the Public Health Service Policy for the Care and Use of Laboratory Animals. Animal care was provided in accordance with the procedures outlined in the “Guide for Care and Use of Laboratory Animals (National Research Council; 1996; National Academy Press; Washington DC). All animal experiments were conducted in accordance with NIH-approved protocols and guidelines.

### Animals and Genotyping

Mice carrying the *Vhl* alleles flanked by loxP sites [25] were re-derived in C57BL/6, A/J and Balb/C backgrounds before crossing with different Cre transgenic mice. The Pdx1-Cre transgenic mice were a kind gift from Dr. D. Melton [37], and the Rip-Cre (B6.Cg-Tg (Ins2-cre)25Mgn/J) transgenic mice were purchased from The Jackson Laboratory (stock number: 003573). The Glu-Cre transgenic mice were generated utilizing the pBKCMV/Glu-CreGH plasmid kindly provided by Dr. M. Magnuson. Briefly, the plasmid fragment containing 2.3 kb of rat glucagon promoter upstream of the Cre-recombinase was isolated by SalI and NotI digestion prior to injection to generate transgenic founder lines. Tissue expression of Glu-Cre was evaluated in offspring from crosses of the Glu-Cre transgenic mice with Z/AP reporter mice [34]. Detailed analysis of the Glu-Cre transgenic mice will be described elsewhere. Cre recombinase expression in tissues of Z/AP mice will delete the floxed *LacZ* expression cassette upstream of the *hPLAP* gene, allowing *hPLAP* expression and detection by standard staining techniques. Tissue sections were counterstained with nuclear fast red (Sigma, St. Louis, MO).

All mice were genotyped by PCR using DNA isolated from tail snips. Tails were digested overnight in 150 µl of DirectPCR Lysis buffer (Viagen Biotech, Los Angeles, CA) following manufacturer's protocol, and used directly for PCR. Cells collected from laser capture microdissection (LCM) and flow cytometry were digested overnight in buffer (100 mM Tris-Cl pH8.0, 50 mM EDTA, 0.2% SDS, 200 mM NaCl) containing fresh proteinase K (1 mg/ml). Genomic DNA was isolated following a standard salt and ethanol precipitation protocol. DNA concentration was determined using NanoDrop ND-1000 (NanoDrop, Wilmington, DE), and equal amounts of DNA were used for PCR analysis. The annealing temperature for Pdx1-Cre, Glu-Cre, and β-globin was 55°C. Primers for Pdx1-Cre (forward: 5′-TTGAAACAAGTGCAGGTGTTCG; reverse: 5′-CCTGAAGATATAGAAGATAATCG), and for Glu-Cre (forward: 5′-AAAATGCAGGCAGATGAGCA; reverse: 5′-CAGGCTGTTGGCGAAGACA), and for β-globin (forward: 5′-CCAATCTGCTCACACAGGATAGAGAGGGCAGG; reverse: 5′-CCTTGAGGCTGTCCAAGTGATTCAGGCCATCG) were utilized to generate 500 bp, 401 bp, and 494 bp PCR products, respectively. The PCR conditions for detecting floxed and deleted *Vhl* alleles have been described [48].

### Animal Tissue Collection and Processing

The pancreatic tissue was processed for frozen histological analysis by embedding tissues in Tissue-Tek OCT freezing medium, and for formalin-fixed paraffin embedding (FFPE). Frozen (10–20 µm) and FFPE pancreas sections (5 µm) were routinely stained with Mayer's hematoxylin and eosin (H&E) for histopathological analysis. For all histological analysis, Cre;Vhl f/f mice were compared with age-matched controls genotypes of Cre, Vhl f/f, Vhl f/+ and Cre;Vhl f/+ mice. Only representative control genotypes are shown in figures. For whole mount mouse pup analysis, a sagittal incision was made to allow formalin fixation to penetrate internal organs. Mouse pups were divided into two parts via the sagittal incision prior to the paraffin embedding process.

FITC-lectin perfusion to visualize the blood vasculature was performed as described [49]. Briefly mice were injected intravenously via tail vein with 50 ug of FITC-labeled lectin (*Lycopersicon esculentum*, Vector Laboratories, Burlingame, CA), which was allowed to circulate for 3 minutes. Then mice were euthanized via cervical dislocation, and the pancreas was removed. All procedures involving mice were performed with approval by the NIH Institutional Animal Care and Use Committee.

### Islet Isolation, Dissociation, and Flow Cytometry

Mice were perfused with FITC-lectin as described above prior to being euthanized. Pancreatic islets were isolated by standard techniques, with minor adaptations. Briefly, pancreata were inflated via bile duct cannulation and retrograde pancreatic duct injection of 3–4 ml of ice-cold collagenase type XI (1 mg/ml in HBSS, Sigma, St. Louis, MO). Following digestion (37°C, 14 min), pancreata were dispersed by gently aspirating through a 14G needle, then filtered through a metal strainer (0.8 mm). Pancreas suspensions were then subjected to buoyant density gradient centrifugation (14–15% Optiprep, Accurate Chemicals, Westbury, NY), followed by handpicking with great care to collect all visible islets.

Isolated islets were dissociated into single cell suspensions by careful pipeting after washing in 2 mM EDTA/PBS, and incubation for 10 min at room temperature in Ca^2+^ free phosphate buffered saline (PBS) supplemented with 0.025% trypsin. Dissociated islet cells were immediately fixed and permeabilized in 4% paraformaldehyde (PFA) (Electron Microscopy Sciences, Hatfield, PA), 0.1% saponin, (Fluka Chemicals, Switzerland) in PBS for 30 min at room temperature. After removing PFA by washing in 0.1 saponin/1% bovine serum albumin (BSA)/PBS, islet cells were stained intracytoplasmically for 30 min with antibodies against insulin (1∶300, guinea pig, DAKO, Carpinteria, CA). After washing, cells were stained in a second staining step with an glucagon monoclonal antibody (clone K79bB10, Sigma, St. Louis, MO) using Zenon (pre)labeling technology for mouse IgG1 (Pacific Blue, Invitrogen, Carlsbad, CA), and an highly cross-absorbed, second-step polyclonal antibodies (pAb), anti-guinea pig-Cy5 (Jackson ImmunoResearch, West Grove, PA). After the final wash in 1%BSA/saponin, cells were post-fixed in 1% PFA, and concomitantly 4-way sorted for insulin positive, glucagon positive, FITC-lectin positive and unstained samples using a FACSAria cell sorter with Diva software (BD Biosciences, San Jose, CA). Electronic gating was set to include viable cells on the basis of forward scatter versus side scatter while the doublet-exclusion gating setup was applied to eliminate non-dissociated islet cell couplets on the basis of pulse width versus total signal area (linear scale). Sorted islet cell subsets were washed in PBS and kept frozen at −70°C until DNA extraction.

### Growth Hormone Measurements

Whole blood was collected via orbital or mandible bleed to isolate serum. Growth hormone levels were measured with the Mouse/Rat Growth Hormone ELISA (Diagnostic Systems Labroatories, Inc., Webster, TX) according to the manufacturer's instructions.

### Glucose Measurements

Mice underwent a 24 hour fast prior to collecting whole blood via a tail snip. Blood glucose was measured using a glucometer (Ascensia Contour, Bayer HealthCare, Mishawaka, IN).

### Immuno-Staining for Histological Analysis

For immuno-histochemical staining, monoclonal mouse anti-human CD31 (1∶25, DAKO clone JC70A, Carpinteria, CA) antibodies, and a cocktail of mouse-anti-cytokeratin, or MAK6 antibodies (predilute, Invitrogen, Carlsbad, CA) were used. The Universal DakoCytomation Labelled Streptavidin-Biotin2 System, Horseradish Peroxidase (LSAB2 System, HRP) followed by the additiona of DAB chromogen was utilized for antigen detection. Sections were counterstained in Mayer's hematoxylin, mounted and photographed using a Zeiss microscope.

For immuno-staining of FITC-lectin injected pancreas, frozen sections were briefly fixed in 4% paraformaldehyde, washed in PBS, and incubated in blocking buffer (5% normal goat serum/2.5% BSA in PBS). Primary antibodies guinea pig anti-swine insulin (1∶500, DAKO, Carpinteria, CA) and rabbit anti-cytokeratin 18 (1∶50, Santa Cruz Biotechnology, Santa Cruz, CA) were diluted in 0.5× blocking buffer. Sections were incubated with primary antibody overnight at 4°C in a humidified chamber followed by brief washes in PBS. Secondary antibodies Alexa Fluor 594 or 633-conjugated anti-guinea pig and Alexa Fluor 594 -conjugated anti-rabbit (1∶200; Invitrogen, Carlsbad, CA) were applied to sections. After incubation at room temperature for one hour, the fluorescently stained sections were washed several times in PBS, cover slipped using mounting medium with DAPI (Vector Laboratories, Burlingame, CA), and visualized using either a Zeiss Axiovert fluorescence microscope (Carl Zeiss MicroImaging, Thornwood, NY).

### RNA collection and Quantitative Real-Time PCR

Islet RNA was collected from frozen pancreas sections on slides by microdissection and extracted using the PicoPure RNA Isolation kit (Arcturus, Mountain View, CA). RNA concentration was determined by NanoDrop ND-1000 (NanoDrop, Wilmington, DE), and equal amounts (1.4 µg) of total RNA was used to generate cDNA. Reverse transcription and quantitative PCR was performed as described previously [50]. TaqMan primers and probes were purchased from Applied Biosystems (Foster City, CA): Gapdh (Mm99999915_g1), Hif1α (Mm00468878_m1), Hif2α/Epas1 (Mm0438717_m1).

### Hif1α ELISA

Total pancreatic protein lysate was harvested by sonicating pancreas on ice in RIPA buffer (Pierce, Rockford, IL) containing 1× Complete Protease Inhibitor Cocktail (Roche, Nutley, NJ). Protein concentration was determined with the Quick Start Bradford Protein Assay (Bio-Rad Laboratories, Hercules, CA). Mouse Hif1α protein was measured using Surveyor IC human/mouse total Hif1α immunoassay according to manufacturer's protocols (R&D Systems, Minneapolis, MN).

### Statistical Analysis

Statistical analysis was performed using GraphPad Prism v.5.01 and Microsoft Excel. The distributions of genotypes after inter-breeding of Cre;Vhl f/+ mice were compared to the expected Mendelian distributions with Chi-square-test. Chi-square and p-value (2 degrees of freedom) are indicated for each strain and each postnatal time point in Tables. Mouse body weight, serum growth hormone levels, and fasted blood glucose levels were evaluated with one-way ANOVA with multiple comparisons testing. Expression data of Hif1α and Hif2α were analyzed with Mann-Whitney (unpaired, nonparametric) t-test. A p value less than (<) 0.05 was considered statistically significant.

## Supporting Information

Figure S1 A. H&E staining of representative Rip-Cre and Rip-Cre;Vhl f/f pancreas at 15 months of age. Islets are as indicated (Is), and images are taken at 100×. B. Genotyping PCR to determine Vhl allele status using genomic DNA isolated from exocrine (exo) and endocrine (endo) pancreas in Rip-Cre;Vhl f/f and Rip-Cre mice at 12 months of age.
(2.54 MB TIF)
