# Supplementary figures and images for: Deciphering von Hippel-Lindau (VHL/Vhl)-Associated Pancreatic Manifestations by Inactivating Vhl in Specific Pancreatic Cell Populations
Source: PLoS One. 2009 Apr 2;4(4):e4897. doi: 10.1371/journal.pone.0004897 (PMC2660574; doi:10.1371/journal.pone.0004897)

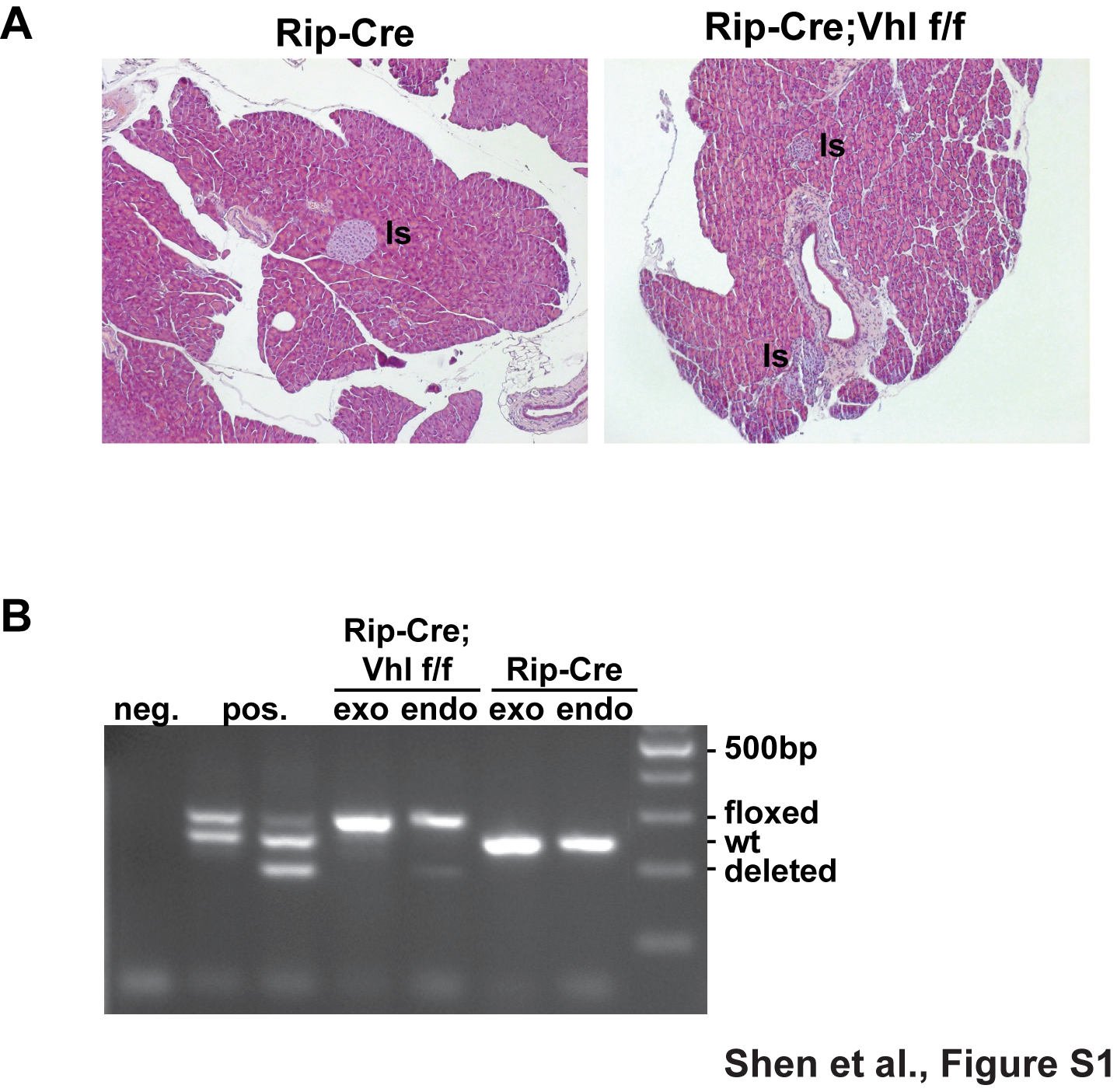

Supplement: Figure S1 — A. H&E staining of representative Rip-Cre and Rip-Cre;Vhl f/f pancreas at 15 months of age. Islets are as indicated (Is), and images are taken at 100×. B. Genotyping PCR to determine Vhl allele status using genomic DNA isolated from exocrine (exo) and endocrine (endo) pancreas in Rip-Cre;Vhl f/f and Rip-Cre mice at 12 months of age. (2.54 MB TIF) [file pone.0004897.s001.tif]
